# Effectiveness of interactive dashboards to optimise prescribing in primary care: a protocol for a systematic review

**DOI:** 10.12688/hrbopenres.13909.1

**Published:** 2024-07-03

**Authors:** Patrick Moynagh, Áine Mannion, Ashley Wei, Barbara Clyne, Frank Moriarty, Caroline McCarthy

**Affiliations:** 1 Department of General Practice, RCSI University of Medicine and Health Sciences, Dublin 2, Ireland; 2 Department of Public Health & Epidemiology, School of Population Health, RCSI University of Medicine and Health Sciences, Dublin 2, Ireland; 3 School of Pharmacy and Biomolecular Sciences, RCSI University of Medicine and Health Sciences, Dublin 2, Ireland

**Keywords:** Interactive dashboards, potentially inappropriate prescribing, audit and feedback, preventable drug related morbidity, polypharmacy

## Abstract

**Introduction:**

Advances in therapeutics and healthcare have led to a growing population of older people living with multimorbidity and polypharmacy making prescribing more challenging. Most prescribing occurs in primary care and General Practitioners (GPs) have expressed interest in comparative feedback on their prescribing performance. Clinical decision support systems (CDSS) and audit and feedback interventions have shown some impact, but changes are often short-lived. Interactive dashboards, a novel approach integrating CDSS and audit and feedback elements, offer longitudinal updated data outside clinical encounters. This systematic review aims to explore the effectiveness of interactive dashboards on prescribing-related outcomes in primary care and examine the characteristics of these dashboards.

**Methods:**

This protocol was prospectively registered on PROSPERO (CRD42023481475) and reported in line with PRISMA-P guidelines. Searches of PubMed, EMBASE, Medline, PsychINFO, CINAHL, Scopus, the Cochrane Library, and grey literature, including trial registries were performed to identify interventional studies (randomised and non-randomised) that assess the effectiveness of interactive dashboards on prescribing related outcomes. The search will be supplemented by searching references of retrieved articles with the use of an automated citation chaser. Identified records will be screened independently by two reviewers and data from eligible studies extracted using a purposely developed data extraction tool. We will narratively summarise the intervention types and those associated with improvements in prescribing outcomes. A quantitative synthesis will be carried out if a sufficient number of homogenous studies are identified. Methodological quality will be assessed by two reviewers using the Cochrane Effective Practice and Organisation of Care risk assessment tool.

**Discussion:**

This systematic review will explore the effect of interactive dashboards on prescribing related outcome measures in primary care and describe the characteristics of interactive dashboards. This research may inform future intervention development and shape policymaking particularly in the context of ongoing and planned developments in e-prescribing infrastructure.

## Introduction

Advances in therapeutics and chronic disease management mean there is a growing population of older people living with multimorbidity and polypharmacy ^1^. While polypharmacy is often necessary and appropriate, it is associated with adverse events, and it is estimated that almost 9% of emergency department admissions in older people are due to preventable drug related morbidity (PDRM) ^2^. Adverse drug reactions (ADRs) are the third most common type of reported adverse event in the Irish health care system ^3^. A recently published prospective cohort study estimated that one in four older people experienced an ADR ^4^. Most prescribing occurs in primary care ^5^ and qualitative data from general practitioners (GPs) indicates prescribing has become more challenging, particularly for patients with multimorbidity and polypharmacy ^6^. Irish GPs in a nationwide cluster randomised controlled trial (RCT) evaluating a deprescribing intervention, viewed participation as an opportunity to review their prescribing practices and were interested in getting performance feedback ^7^.

Primary care prescribers receive feedback on their prescribing through various means, such as clinical decision support systems (CDSS) and audit and feedback. CDSS are real-time, electronic tools that provide prescribers with knowledge and person-specific information at the point of care, which supplement decision-making processes ^8^. CDSS are embedded in clinical software and typically appear as “alerts” for the prescriber. However problems such as interrupting work flow and too many alerts can cause “alert fatigue” resulting in the user ignoring recommendations ^9^. Evidence suggests CDSS probably have a small effect on practitioner performance but the effect on patient reported and clinical outcomes is less clear ^10, 11^. Audit and feedback involves retrospectively reviewing clinical performance or practices, enabling peer comparison and social norm feedback and it has been identified as an effective strategy for improving prescribing ^12, 13^. However, audit and feedback data typically provide a snapshot at one time point and therefore improvements may be temporary ^14^. Interactive dashboards combine elements of both CDSS and audit and feedback; the data is longitudinal and updated on an ongoing basis but is outside the clinical encounter. The prescriber can visualise their data graphically and the data can be manipulated and interacted with through various interactive elements, identifying both time trends and comparisons with peers ^15^.

Medicines optimisation interventions that target a heterogonous population often use prescribing-related outcome measures, as clinical outcome’s such as ADRs or unplanned hospital admissions may take time to manifest or reach measurable levels ^16^. Various tools have been developed to assess the quality of prescribing and broadly speaking these can be categorised into two groups: explicit tools and implicit tools. A systematic review published in 2014 identified 46 different explicit and implicit tools that have been developed to assess medication appropriateness ^17^. Examples of explicit measures of medication appropriateness include the United States (US) Beers criteria ^18^ and the European Screening Tool for Older People’s potentially inappropriate Prescriptions (STOPP) criteria ^19^. Multiple observational studies have demonstrated an association between potentially inappropriate prescribing, measured using these indicators, and clinical outcomes such as increased emergency admissions, ADRs and reduced health related quality of life ^20– 22^. In addition, specific research groups have identified high-risk and low-value prescribing criteria and evaluated the effectiveness of interventions utilising these criteria ^23– 25^. For example the Data-driven Quality Improvement in Primary Care (DQIP) intervention included an informatics tool that provided weekly updates of selected high risk prescribing indicators to clinicians, and facilitated medication review by graphically displaying relevant drug history data ^26^. The pharmacist-led information technology intervention (PINCER) was effective at reducing hazardous prescribing, however the effect may have been temporary as the original intervention provided a snapshot of data from the electronic health record ^23^. More recently an interactive dashboard utilizing the PINCER criteria has been developed whereby the user can track their performance across different criteria compared to other practices and over time ^27^, and this intervention resulted in a reduction of potentially hazardous prescribing by 27.9% (95% CI 20.3% to 36.8%, p < 0.001) ^28^.

This systematic review aims to explore the effectiveness of interactive dashboards on prescribing related outcomes in primary care and to describe the characteristics of these interventions with the ultimate aim of informing future intervention development and e-prescribing infrastructure.

## Methods

This systematic review was prospectively registered on PROSPERO (CRD42023481475), it will be conducted in line with guidance set out in the Cochrane Handbook for Systematic Reviews of Interventions ^29^, and reported in adherence to PRISMA-P reporting guidelines ^30^. At the time of writing, the search strategy has been finalised, title and abstract screening has been completed, and full text review is currently in process.

### Search strategy

An information specialist in the host institution’s library with extensive experience in supporting systematic reviews was involved in developing search strategies. A systematic literature search was conducted and included the following databases; PubMed, EMBASE, MEDLINE (OVID), PsycINFO (EBSCOhost), CINAHL (EBSCOhost), Scopus and the Cochrane Library (OVID).

A search of grey literature was conducted by running keyword searches in OpenGrey, CADTH Grey Matters and web-based clinical trial registries. The search was supplemented by searching references of retrieved articles with the use of an automated citation chaser ^31^. No restrictions were placed on language or year of publication. Search terms included “interactive dashboard” and the medical subject heading (MeSH) “clinical audit”, “medical audit”, “benchmarking” and “feedback” and keywords to capture concepts related to providing prescribers with feedback, such as “electronic health record” and “alerts”. See supplementary file 1 for electronic search reports, including the full search terms. 

### Study selection

Identified records were uploaded to Covidence systematic review software and de-duplicated. Reviewers were blinded to minimise potential bias and ensure impartial evaluation of the included studies. Two reviewers independently read the titles/abstracts of identified records and eliminated studies not meeting inclusion criteria. The full text of the remaining studies will be reviewed again by two reviewers who will assess their suitability for inclusion. Disagreement will be resolved through discussion with the wider study group. Eligibility criteria are described in Table 1. All interventional designs will be included including randomised controlled trials (RCTs) (e.g. cluster RCTs, step wedged RCTs and individually randomised RCTs) and non-randomised interventional studies (e.g. interrupted time series design and controlled before and after studies) ^32^.

**Table 1.** Study eligibility criteria.

Criteria	Inclusion	Exclusion
**Population**	Primary care prescribers (e.g. General Practitioners, non-medical prescribers based in primary care such as pharmacists and advanced nurse practitioners)	Primary care prescribers working in a secondary care setting. Dentists
**Intervention**	An interactive dashboard designed to provide feedback on prescribing data to prescribers and including the following characteristics: **Visual display of data**: Data is presented in the form of graphs or tables. **Interactivity**: Allows direct manipulation with visual analytical tools or provides multiple parameters from the dataset, accessible online or via email. **Real-time data**: Offers real-time or relatively contemporaneous data, no older than one year. **Frequent data feedback**: Provides data feedback more than once. **Comparative analysis**: Compares data to peers or set standards.	Simple CDSS interventions Audit and feedback interventions that do not give longitudinal and ongoing feedback
**Comparator**	Usual care	
**Outcomes**	**Primary Outcome:** Prescribing related outcome measures such as implicit/explicit criteria, high-risk or low-value criteria or where relevant prescribing rates (e.g. where a higher rate may reflect lower quality such as benzodiazepine or opioid use).	No prescribing outcomes measured
**Setting**	Primary care (Family practice, general practice)	Studies focused on specialist clinics, nursing homes, hospital based, dental surgeries.
**Study design**	Interventional studies both randomised and non-randomised designs (e.g. Randomised controlled trials, non-randomised controlled trials, controlled before and after studies and interrupted time series)	Systematic reviews, descriptive study designs (e.g., case reports, uncontrolled before and after studies). Letters, commentaries, editorials.
**Publication ** **language**	No language restriction	
**Dates of ** **publication**	No year limitation	

### Data extraction and management

Two review authors will independently extract data using a purposely developed data extraction tool in Covidence, developed with use of the Template for Intervention Description and Replication (TIDieR) checklist ^33^. Extracted data will include study details (e.g. setting, design), population (e.g. GPs), intervention details, comparison group, outcome measures and results. Table 2 outlines example data that may be extracted to describe the intervention using the TIDieR checklist. We will attempt to contact the lead authors of primary studies to locate missing data. Discrepancies will be resolved through discussion and consensus between two reviewers with consultation with a third reviewer if necessary.

**Table 2.** Intervention data extraction using adapted TIDieR checklist.

Description	Detail	Example Intervention
**1. Brief name**	Provide a concise name for the intervention.	"Prescribing Dashboard"
**2. Why**	Rationale or goal of the intervention.	To improve prescribing quality by providing GPs with real-time data on high-risk prescribing for comparative benchmarking against peers.
**3. What (materials ** **and procedures)**	Materials used and procedures for the intervention.	Web-based or practice software-embedded dashboard displaying prescribing quality metrics. GPs access their prescribing metrics on the dashboard at their convenience for self-assessment and benchmarking.
**4. Who provided**	Description of the intervention providers.	Developed by healthcare IT specialists in collaboration with clinicians.
**5. How and where**	Modes and location of delivery.	Delivered via a secure web-based platform or embedded within practice management software, accessible in primary care clinics and offices on various devices.
**6. When and how ** **much**	Frequency and duration.	Dashboards updated monthly with new prescribing data to reflect recent prescribing practices.
**7. Tailoring**	Personalization of the intervention.	Dashboard data tailored to individual prescriber level or practice level, depending on the setting and objectives of the feedback.
**8. Modifications**	Changes made during the study.	N/A or describe any modifications based on user feedback or technological updates.
**9. How well (planned ** **and actual)**	Assessment of adherence or fidelity and actual engagement.	Usage analytics to monitor frequency of dashboard interactions, time spent on the dashboard, and engagement with specific metrics.

***Methodological quality assessment*.** Methodological Quality Assessment will be performed by two reviewers using the Cochrane Effective Practice and Organisation of Care (EPOC) risk of bias tool ^32^. Discrepancies will be resolved through discussion and consensus between two reviewers with consultation with a third reviewer if necessary.

***Analysis*.** We will narratively summarise the intervention types and those associated with improvements in prescribing outcomes. Additionally, we will narratively describe the prescribing related outcomes used by included studies. A quantitative synthesis (i.e. meta-analysis) will be considered if a sufficient number of homogenous studies are identified which examine the same outcome.

If a meta-analysis is conducted, a random-effects model will likely be appropriate given the review question. We will not combine results from different study designs and interventions in an overall meta-analysis. Results will be presented in separate subgroups in the same forest plot (with no summary effect estimate) Heterogeneity will be assessed through a visual assessment and a logic-based assessment of study differences. We will conduct a standard Q-test statistic for heterogeneity and evaluate the heterogeneity via the *I²* statistic, which can be interpreted as the proportion of variability in the meta-analysis due to between study heterogeneity. Funnel plots will explore publication bias if more than ten studies are identified. These plots will help assess the relationship between effect size and study precision.

All missing outcome data for included studies will be recorded on the data extraction form and reported in the risk of bias table. If there is insufficient information on the primary outcomes (due to inability to contact authors, unavailable data), these studies will be reported separately. Reasons for exclusion will be described and included in a supplementary table. As this systematic review is assessing an intervention targeted at primary care prescribers, included studies may have aggregate level patient data, thus it may not be possible to conduct population level subgroup analysis.

## Discussion

With a growing population of older people living with multimorbidity and polypharmacy, prescribing has become more challenging with a greater propensity for adverse outcomes ^6, 16^. PDRM has significant economic and social consequences at both the individual patient-level and for the wider healthcare system ^34^, it is thus vital to develop interventions to support safe and effective prescribing.

Interactive dashboards have become increasingly prevalent in healthcare settings, offering a versatile tool for visualising clinical data across various levels ranging from organisational, physician to patient-focused applications. They have the potential to enhance patient care and safety by providing contemporaneous feedback on potentially suboptimal treatment or care when integrated into clinical record systems ^35^.

Interactive dashboards have demonstrated varied effects on prescribing-related outcomes, such as antibiotic prescribing rates and appropriate statin use ^36, 37^. Current evidence suggests they are most effective when combined with additional strategies which include education and/or behavioural components ^37^. Given the limited number of eligible studies identified in previous reviews, the present systematic review will not restrict its focus to specific medication classes.

### Strengths and limitations

This research will be conducted in line with Cochrane guidance. To increase transparency and reduce the risk of selective reporting this systematic review has been prospectively registered on PROSPERO, and will involve a search of the grey literature and trial registries to reduce the risk of publication bias. Title and abstract screening, full-text review, and methodological quality assessment will be performed by two reviewers working independently and blinded to each other's assessments, thereby minimising the potential for bias and errors. Excluding studies in progress but not yet published may lead to publication bias.

### Potential implications for future research, policy and clinical practice

This research may identify gaps in the current literature and inform future intervention development with respect to how prescribing data may be fed back to prescribers. The findings from this review may inform policies aimed at enhancing or expanding the infrastructure necessary for effective e-prescribing, particularly those focused on optimising prescribing behaviours. In addition, this review will provide prescribers with a synthesised understanding of how interactive dashboards have been used, highlighting their potential benefits and limitations. This may lead to more informed decisions in regards adopting or optimising use of such tools in clinical practice, with the ultimate aim of improving patient safety and reducing medication related harm.

## Data Availability

No underlying data are associated with this article. ***This project contains the following extended data:*** figshare: Supplementary file 1: Electronic search reports https://doi.org/10.6084/m9.figshare.25859506.v1 ^38^ fighare: Supplementary file 2: PRISMA-P 2015 checklist https://doi.org/10.6084/m9.figshare.25887193.v1 ^39^ Data are available under the terms of the Creative Commons Attribution 4.0 International license (CC-BY 4.0) ( https://creativecommons.org/licenses/by/4.0/)

## References

[1] Fortin M Stewart M Poitras ME : A systematic review of prevalence studies on multimorbidity: toward a more uniform methodology. *Ann Fam Med.* 2012;10(2):142–51. 10.1370/afm.1337 22412006 PMC3315131

[2] Oscanoa TJ Lizaraso F Carvajal A : Hospital admissions due to adverse drug reactions in the elderly. A meta-analysis. *Eur J Clin Pharmacol.* 2017;73(6):759–70. 10.1007/s00228-017-2225-3 28251277

[3] Connolly W Rafter N Conroy RM : The Irish National Adverse Event Study-2 (INAES-2): longitudinal trends in adverse event rates in the Irish healthcare system. *BMJ Qual Saf.* 2021;30(7):547–58. 10.1136/bmjqs-2020-011122 33436402 PMC8237194

[4] Doherty AS Boland F Moriarty F : Adverse drug reactions and associated patient characteristics in older community-dwelling adults: a 6-year prospective cohort study. *Br J Gen Pract.* 2023;73(728):e211–e219. 10.3399/BJGP.2022.0181 36823047 PMC9923764

[5] Avery AJ Ghaleb M Barber N : The prevalence and nature of prescribing and monitoring errors in English general practice: a retrospective case note review. *Br J Gen Pract.* 2013;63(613):e543–53. 10.3399/bjgp13X670679 23972195 PMC3722831

[6] Sinnott C Mc Hugh S Browne J : GPs' perspectives on the management of patients with multimorbidity: systematic review and synthesis of qualitative research. *BMJ Open.* 2013;3(9): e003610. 10.1136/bmjopen-2013-003610 24038011 PMC3773648

[7] McCarthy C Pericin I Smith SM : Patient and general practitioner experiences of implementing a medication review intervention in older people with multimorbidity: process evaluation of the SPPiRE trial. *Health Expect.* 2022;25(6):3225–3237. 10.1111/hex.13630 36245339 PMC9700182

[8] Sutton RT Pincock D Baumgart DC : An overview of clinical decision support systems: benefits, risks, and strategies for success. *NPJ Digit Med.* 2020;3(1): 17. 10.1038/s41746-020-0221-y 32047862 PMC7005290

[9] Hayward J Thomson F Milne H : 'Too much, too late': mixed methods multi-channel video recording study of computerized decision support systems and GP prescribing. *J Am Med Inform Assoc.* 2013;20(e1):e76–e84. 10.1136/amiajnl-2012-001484 23470696 PMC3715350

[10] Asmar MLE Dharmayat KI Vallejo-Vaz AJ : Effect of computerised, knowledge-based, clinical decision support systems on patient-reported and clinical outcomes of patients with chronic disease managed in primary care settings: a systematic review. *BMJ Open.* 2021;11(12): e054659. 10.1136/bmjopen-2021-054659 34937723 PMC8705223

[11] Garg AX Adhikari NK McDonald H : Effects of computerized clinical decision support systems on practitioner performance and patient outcomes: a systematic review. *JAMA.* 2005;293(10):1223–38. 10.1001/jama.293.10.1223 15755945

[12] Gould IM Lawes T : Antibiotic stewardship: prescribing social norms. *Lancet.* 2016;387(10029):1699–701. 10.1016/S0140-6736(16)00007-6 26898851

[13] Zeng Y Shi L Liu C : Effects of social norm feedback on antibiotic prescribing and its characteristics in behaviour change techniques: a mixed-methods systematic review. *Lancet Infect Dis.* 2023;23(5):e175–e184. 10.1016/S1473-3099(22)00720-4 36521504

[14] Ivers N Jamtvedt G Flottorp S : Audit and feedback: effects on professional practice and healthcare outcomes. *Cochrane Database Syst Rev.* 2012; (6): Cd000259. 10.1002/14651858.CD000259.pub3 22696318 PMC11338587

[15] Jeffries M Gude WT Keers RN : Understanding the utilisation of a novel interactive electronic medication safety dashboard in general practice: a mixed methods study. *BMC Med Inform Decis Mak.* 2020;20(1): 69. 10.1186/s12911-020-1084-5 32303219 PMC7164282

[16] McCarthy C Flood M Clyne B : Medication changes and potentially inappropriate prescribing in older patients with significant polypharmacy. *Int J Clin Pharm.* 2023;45(1):191–200. 10.1007/s11096-022-01497-2 36385206

[17] Kaufmann CP Tremp R Hersberger KE : Inappropriate prescribing: a systematic overview of published assessment tools. *Eur J Clin Pharmacol.* 2014;70(1):1–11. 10.1007/s00228-013-1575-8 24019054

[18] Fick DM Semla TP Beizer J : American geriatrics society 2015 updated beers criteria for potentially inappropriate medication use in older adults. *J Am Geriatr Soc.* 2015;63(11):2227–46. 10.1111/jgs.13702 26446832

[19] O'Mahony D Cherubini A Guiteras AR : STOPP/START criteria for potentially inappropriate prescribing in older people: version 3. *Eur Geriatr Med.* 2023;14(4):625–632. 10.1007/s41999-023-00777-y 37256475 PMC10447584

[20] Wallace E McDowell R Bennett K : Impact of potentially inappropriate prescribing on adverse drug events, health related quality of life and emergency hospital attendance in older people attending general practice: a prospective cohort study. *J Gerontol A Biol Sci Med Sci.* 2016. 10.1093/gerona/glw140 PMC5233913 27466245

[21] Cahir C Moriarty F Teljeur C : Potentially inappropriate prescribing and vulnerability and hospitalization in older community-dwelling patients. *Ann Pharmacother.* 2014;48(12):1546–54. 10.1177/1060028014552821 25248541

[22] Cahir C Bennett K Teljeur C : Potentially inappropriate prescribing and adverse health outcomes in community dwelling older patients. *Br J Clin Pharmacol.* 2014;77(1):201–10. 10.1111/bcp.12161 23711082 PMC3895361

[23] Avery AJ Rodgers S Cantrill JA : A pharmacist-led information technology intervention for medication errors (PINCER): a multicentre, cluster randomised, controlled trial and cost-effectiveness analysis. *Lancet.* 2012;379(9823):1310–9. 10.1016/S0140-6736(11)61817-5 22357106 PMC3328846

[24] Dreischulte T Donnan P Grant A : Safer prescribing--a trial of education, informatics, and financial incentives. *N Engl J Med.* 2016;374(11):1053–64. 10.1056/NEJMsa1508955 26981935

[25] Radomski TR Decker A Khodyakov D : Development of a metric to detect and decrease low-value prescribing in older adults. *JAMA Netw Open.* 2022;5(2): e2148599. 10.1001/jamanetworkopen.2021.48599 35166780 PMC8848205

[26] Grant AM Guthrie B Dreischulte T : Developing a complex intervention to improve prescribing safety in primary care: mixed methods feasibility and optimisation pilot study. *BMJ Open.* 2014;4(1): e004153. 10.1136/bmjopen-2013-004153 24448848 PMC3902335

[27] Williams R Keers R Gude WT : SMASH! The salford medication safety dashboard. *J Innov Health Inform.* 2018;25(3):183–93. 10.14236/jhi.v25i3.1015 30398462

[28] Peek N Gude WT Keers RN : Evaluation of a pharmacist-led actionable audit and feedback intervention for improving medication safety in UK primary care: an interrupted time series analysis. *PLoS Med.* 2020;17(10): e1003286. 10.1371/journal.pmed.1003286 33048923 PMC7553336

[29] Higgins JPT Thomas J Chandler J : Cochrane handbook for systematic reviews of interventions.version 6.4 (updated August 2023): Cochrane;2023. Reference Source

[30] Moher D Shamseer L Clarke M : Preferred reporting items for systematic review and meta-analysis protocols (PRISMA-P) 2015 statement. *Syst Rev.* 2015;4(1): 1. 10.1186/2046-4053-4-1 25554246 PMC4320440

[31] Haddaway NR Grainger MJ Gray CT : citationchaser: an R package for forward and backward citations chasing in academic searching.0.0.3 ed2021. 10.1002/jrsm.1563 35472127

[32] Cochrane Effective Practice and Organisation of Care (EPOC): What study designs can be considered for inclusion in an EPOC review and what should they be called? 2017; 13/03/2024. Reference Source

[33] Hoffmann TC Glasziou PP Boutron I : Better reporting of interventions: template for intervention description and replication (TIDieR) checklist and guide. *BMJ.* 2014;348: g1687. 10.1136/bmj.g1687 24609605

[34] Hodkinson A Tyler N Ashcroft DM : Preventable medication harm across health care settings: a systematic review and meta-analysis. *BMC Med.* 2020;18(1): 313. 10.1186/s12916-020-01774-9 33153451 PMC7646069

[35] Dowding D Randell R Gardner P : Dashboards for improving patient care: review of the literature. *Int J Med Inform.* 2015;84(2):87–100. 10.1016/j.ijmedinf.2014.10.001 25453274

[36] Xie CX Chen Q Hincapie CA : Effectiveness of clinical dashboards as audit and feedback or clinical decision support tools on medication use and test ordering: a systematic review of randomized controlled trials. *J Am Med Inform Assoc.* 2022;29(10):1773–85. 10.1093/jamia/ocac094 35689652 PMC9471705

[37] Garzón-Orjuela N Parveen S Amin D : The effectiveness of interactive dashboards to optimise antibiotic prescribing in primary care: a systematic review. *Antibiotics (Basel).* 2023;12(1):136. 10.3390/antibiotics12010136 36671337 PMC9854857

[38] Supplementary file 1 Electronic search reports. *figshare.* 2024. 10.6084/m9.figshare.25859506.v1

[39] Supplementary file 2 PRISMA-P 2015 checklist. *figshare.* 2024. 10.6084/m9.figshare.25887193.v1

