# Supplementary material for: Effectiveness of interactive dashboards to optimise prescribing in primary care: a protocol for a systematic review
Source: HRB Open Res. 2025 Feb 19;7:44. Originally published 2024 Jul 3. [Version 2] doi: 10.12688/hrbopenres.13909.2 (PMC11808840; doi:10.12688/hrbopenres.13909.2)
Supplement: Peer review history

# Reviewer report: Reviewer response for version 2

**DOI:** 10.21956/hrbopenres.15476.r45918

**Published:** 2025-02-27

**Authors:** Rainer Tan, Nina Emery

**Affiliations:** 1 University of Lausanne, Lausanne, Switzerland

We thank the authors for their answers. The revised manuscript shows more comprehensiveness and clarity, and all our comments have been addressed. Particularly, the outcomes of interest and their prioritization is clearer, as well as the distinction between explicit and implicit tools. All the minor issues have also been correctly addressed. We have no further comments.

Is the study design appropriate for the research question?

Partly

Is the rationale for, and objectives of, the study clearly described?

Partly

Are sufficient details of the methods provided to allow replication by others?

Yes

Are the datasets clearly presented in a useable and accessible format?

Yes

Reviewer Expertise:

Digital Health, antibiotic stewardship

We confirm that we have read this submission and believe that we have an appropriate level of expertise to confirm that it is of an acceptable scientific standard.

# Reviewer report: Reviewer response for version 2

**DOI:** 10.21956/hrbopenres.15476.r45919

**Published:** 2025-02-20

**Authors:** Heike Vornhagen

**Affiliations:** 1 University of Galway, Galway, Ireland

I am happy with the revised version and have no further comments to make.

Is the study design appropriate for the research question?

Yes

Is the rationale for, and objectives of, the study clearly described?

Partly

Are sufficient details of the methods provided to allow replication by others?

Yes

Are the datasets clearly presented in a useable and accessible format?

Not applicable

Reviewer Expertise:

Dashboards, Data Visualisation, UX Design

I confirm that I have read this submission and believe that I have an appropriate level of expertise to confirm that it is of an acceptable scientific standard.

# Reviewer report: Reviewer response for version 2

**DOI:** 10.21956/hrbopenres.15476.r45916

**Published:** 2025-02-20

**Authors:** Augustino Mwogosi

**Affiliations:** 1 The University of Dodoma, Dodoma, Dodoma Region, Tanzania

The revised manuscript demonstrates significant improvements in clarity, methodological transparency, and justification for the study. The authors have effectively addressed the primary concerns and enhanced the protocol's overall rigour. The introduction has been strengthened by providing a clearer rationale for conducting the systematic review, particularly about medication-related harm, polypharmacy, and the challenges of prescribing in primary care. Including additional references and clarifying key concepts, such as the distinction between implicit and explicit medication appropriateness tools, further contributes to the manuscript’s clarity.

 Methodological improvements are evident in the study selection criteria, which now explicitly state that all patient cohorts within primary care settings will be considered. This revision resolves prior concerns about whether the review was specifically focused on older adults or had a broader scope. The authors have also refined the analysis section by specifying the criteria for determining study homogeneity in meta-analysis, which includes study design, outcome definitions, and measurement levels. This level of detail ensures a more transparent and reproducible methodology. Moreover, the clarification that two independent reviewers will conduct full-text screening further strengthens the rigour of the study selection process.

 The manuscript now provides a more precise definition of “effectiveness” concerning interactive dashboards, linking it to prescribing-related outcomes such as potentially inappropriate prescribing (PIP) and adherence to guidelines. Sensitivity analyses are now explicitly discussed, although no specific subgroup analyses are planned due to the anticipated aggregate nature of the data. While this limitation is acknowledged, the authors may consider briefly mentioning whether any exploratory subgroup analyses (e.g., based on intervention type or setting) could be considered if the data allow. This minor addition would further strengthen the interpretability of the findings.

Is the study design appropriate for the research question?

Yes

Is the rationale for, and objectives of, the study clearly described?

Partly

Are sufficient details of the methods provided to allow replication by others?

Yes

Are the datasets clearly presented in a useable and accessible format?

Partly

Reviewer Expertise:

The core areas of research include Health Informatics and Decision-Support Systems, which focus on optimising the implementation and use of Electronic Health Record (EHR) systems to improve clinical decision-making in primary healthcare. Research also explores the integration of generative AI in EHR systems, particularly in low-resource settings.

I confirm that I have read this submission and believe that I have an appropriate level of expertise to confirm that it is of an acceptable scientific standard.

# Reviewer report: Reviewer response for version 1

**DOI:** 10.21956/hrbopenres.15255.r43666

**Published:** 2024-12-28

**Authors:** Rainer Tan, Nina Emery

**Affiliations:** 1 University of Lausanne, Lausanne, Switzerland

The authors highlight the challenge of appropriate prescribing for patients with multimorbidity and polypharmacy, which is a growing problem in the context of populational aging. More specifically, they focus on primary care, where the majority of prescribing takes place. Interactive dashboards are identified as a potential solution to help primary healthcare workers improve prescribing safety. They propose a protocol for a systematic review with the aim of evaluating the effectiveness of interactive dashboards on prescribing outcomes. Overall, the protocol is clear, well-developed, and aligned with the PRISMA-P and Cochrane guidelines.

 However, some changes could improve overall clarity and completeness:

 Major issue:

1. In line with section 13 of PRISMA-P guidelines, more details on the different outcomes of interest, the type of measures and their prioritization should be provided. It is unclear if the outcomes listed in table 1 are an exhaustive list of the outcome of interest in the review, or the outcome upon which studies will be selected for the review. Moreover, in line with Cochrane guidelines ( **MECIR Box** **3.2.d** C17), authors should define which measure of prescribing quality will be prioritized, if multiple measures are reported (eg, explicit vs implicit criteria) or provide a rationale if not possible. Finally clarifying the difference between implicit and explicit tools would add clarity.

Minor issues:

1. Last paragraph of introduction: the concept of “effectiveness” of interactive dashboards on prescribing related outcomes would benefit from further clarification. How is effectiveness defined and measured? Is it, for example, the dashboards’ effect on the rate of prescriptions aligned with Beers or STOPP criteria?
2. Study selection: In line with section 8 of PRISMA-P guidelines, the authors report the study characteristics to be used as eligibility criteria for the review, in a clear manner as part of a table (table 1), but the described population of interest would benefit from more details. Indeed, in the description of the population of interest, the authors mention primary care prescribers. However, there is no mention of the type of patients targeted by the intervention. Given the focus given in the introduction on the elderly population, and given that tools such as Beers and STOPP are designed for elderly patients, does the review focus on geriatric patients? Children? Studies looking at patients with particular illnesses (e.g. COVID-19, HIV)?
3. Study selection: The authors state that full text will be reviewed by two reviewers, however not completely clear whether this will be done independently or subsequently.
4. Analysis: In line with PRISMA-P section 15a, the protocol is clear that a meta-analysis will be performed if there are sufficient studies examining the same outcome, but it is unclear what other criteria will be used to define «homogenous studies».
5. Analysis: Will any subgroup or sensitivity analyses be performed? If so, they should ideally be described.

Is the study design appropriate for the research question?

Partly

Is the rationale for, and objectives of, the study clearly described?

Partly

Are sufficient details of the methods provided to allow replication by others?

Yes

Are the datasets clearly presented in a useable and accessible format?

Yes

Reviewer Expertise:

Digital Health, antibiotic stewardship

We confirm that we have read this submission and believe that we have an appropriate level of expertise to confirm that it is of an acceptable scientific standard, however we have significant reservations, as outlined above.

# Response

**Published:** 2025-02-06

**Authors:** Patrick Moynagh

**Affiliations:** Department of General Practice, RCSI University of Medicine and Health Sciences, Dublin, Ireland

Thank you very much for reviewing our manuscript. Your comments are very helpful and appreciated. Please see our replies below. 

**Major issue:**

**In line with section 13 of PRISMA-P guidelines, more details on the different outcomes of interest, the type of measures and their prioritization should be provided. It is unclear if the outcomes listed in table 1 are an exhaustive list of the outcome of interest in the review, or the outcome upon which studies will be selected for the review. Moreover, in line with Cochrane guidelines (MECIR Box 3.2.d C17), authors should define which measure of prescribing quality will be prioritized, if multiple measures are reported (eg, explicit vs implicit criteria) or provide a rationale if not possible. Finally clarifying the difference between implicit and explicit tools would add clarity.**

 In the protocol, we intentionally kept the definition of prescribing-related outcome measures broad, encompassing measures such as utilisation patterns and prescribing quality assessed through explicit and/or implicit criteria. This approach was informed by scoping searches, which suggested that explicit criteria and utilisation patterns were likely to be the predominant method in these studies which typically analyse large populations at aggregate levels. While implicit criteria were not explicitly excluded, they did not emerge in the included studies during the review process, likely due to the operational constraints of applying implicit tools in such contexts. We acknowledge that further detail on the prioritisation of outcomes would be helpful and have now added  clarification that while both explicit and implicit criteria were considered, we anticipated, based on our scoping searches, that explicit criteria and utilisation patterns would feature more prominently. Additionally, we have expanded on the distinction between explicit and implicit tools in the revised text to provide clarity. We have also clarified that all prescribing-related outcome measures that meet our inclusion criteria will be included.

 We have updated the manuscript as follows:

**Introduction:** *“Explicit tools are focused on drugs, measuring how they fit pre-defined criteria whereas implicit tools are based on clinical guidelines and clinical evaluation criteria”*

**Methods: Study Selection** ***: **“All outcome measures detailed in Table 1 will be considered, we anticipate however, based on scoping searches, that explicit criteria and utilisation patterns will feature more prominently.”*

**Minor Issues:**

**1) Last paragraph of introduction: the concept of “effectiveness” of interactive dashboards on prescribing related outcomes would benefit from further clarification. How is effectiveness defined and measured? Is it, for example, the dashboards’ effect on the rate of prescriptions aligned with Beers or STOPP criteria?**

 Thank for you highlighting this point. the introduction paragraph has been updated as follows:

*“In line with national and international campaigns to reduce medication-related harm and recent developments in e-prescribing infrastructure, this systematic review aims to explore the effectiveness of interactive dashboards in improving prescribing-related outcomes in primary care. These outcomes include potentially inappropriate prescribing (PIP) and drug utilisation rates (e.g., reducing prescribing volumes where lower rates are preferable or optimising prescribing patterns in line with guidelines). Additionally, it aims to describe the characteristics of these interventions to inform future intervention development and e-prescribing infrastructure.”*

**2) Study selection: In line with section 8 of PRISMA-P guidelines, the authors report the study characteristics to be used as eligibility criteria for the review, in a clear manner as part of a table (table 1), but the described population of interest would benefit from more details. Indeed, in the description of the population of interest, the authors mention primary care prescribers. However, there is no mention of the type of patients targeted by the intervention. Given the focus given in the introduction on the elderly population, and given that tools such as Beers and STOPP are designed for elderly patients, does the review focus on geriatric patients? Children? Studies looking at patients with particular illnesses (e.g. COVID-19, HIV)?**

 We agree this needs clarification. As this intervention is targeted at clinicians but outcomes measured on patients/populations there are two populations of interest. We have clarified this within Table 1.  

**3) Analysis: In line with PRISMA-P section 15a, the protocol is clear that a meta-analysis will be performed if there are sufficient studies examining the same outcome, but it is unclear what other criteria will be used to define «homogenous studies»**.

 In the protocol, we outlined that meta-analysis would be performed if there were sufficient studies examining the same outcome. To further clarify, we defined homogeneity based on several key factors:

**Study Design**: Studies needed to share a similar design (e.g., observational studies, interventional studies) to ensure comparability.

**Outcome Definition**: Outcomes needed to be defined and measured in a similar way across studies.

**Level of Measurement**: Outcomes measured at comparable levels, such as practice-level prescribing rates versus patient-level prescribing rates, were grouped separately to ensure consistency.

 These criteria aimed to balance comparability while maintaining a sufficiently broad scope to include a wide range of prescribing-related outcomes. Where studies differed in any of these aspects, meta-analysis was not performed, and results were synthesised narratively.

 We have updated the manuscript as follows:

*“We will narratively summarise the intervention types and those associated with improvements in prescribing outcomes. Additionally, we will narratively describe the prescribing related outcomes used by included studies. A quantitative synthesis (i.e. meta-analysis) will be considered if a sufficient number of homogenous studies are identified which examine the same outcome. We defined homogeneity based on several key factors, (i) study design (ii) outcome definition (iii) level of measurement”*

**4) Analysis: Will any subgroup or sensitivity analyses be performed? If so, they should ideally be described.**

 Given the nature of the intervention we anticipated that outcomes will be measured at aggregate level, thus population level subgroup analyses were not planned as they were unlikely to be feasible.

 We have noted in the last sentence of the analysis:

*“As this systematic review is assessing an intervention targeted at primary care prescribers, included studies may have aggregate level patient data, thus it may not be possible to conduct population level subgroup analysis.”*

 Sensitivity analysis can be performed. The manuscript was updated as follows. 

*“As we believe that heterogeneity exists regardless of whether we happen to detect it using a statistical test, we will focus less on significance tests and instead further investigate the sources and impact of the heterogeneity (e.g Risk of Bias) through sensitivity analysis.  In the event of substantial clinical or methodological heterogeneity, we will not report study results as pooled effect estimates and will synthesise study findings using the approach suggested in the Synthesis Without Meta‐analysis (SWIM) guidance.”*

**5) Study selection: The authors state that full text will be reviewed by two reviewers, however not completely clear whether this will be done independently or subsequently**

 Thank you for highlighting this omission in the manuscript. The manuscript was updated as follows:

*“Two reviewers independently read the titles/abstracts of identified records and eliminated studies not meeting inclusion criteria. The full text of the remaining studies will be reviewed again independently by two reviewers who will assess their suitability for inclusion.”*

# Reviewer report: Reviewer response for version 1

**DOI:** 10.21956/hrbopenres.15255.r43664

**Published:** 2024-12-26

**Authors:** Heike Vornhagen

**Affiliations:** 1 University of Galway, Galway, Ireland

A potentially interesting study. However, it is unclear to me if the focus is on prescribing in primary care generally as indicated in the title, or on older people and multi-morbidity / polypharmacy. This confusion is carried forward into the introduction which starts with CDM and older people but then exclusively focuses on dashboards, feedback and prescribing with no further mention of older people. I would suggest to clarify the focus of the systematic review and update the article accordingly.

 This paper describes a protocol for carrying out a systematic review of interactive dashboards and prescribing in primary care. It gives an overview of the context for the research and details the search strategy. However, the context veers between care of older people especially regarding multi-morbidity and polypharmacy and the use of interactive dashboards to improve prescribing. It is not clear if there will be a link established in the full review, however, as the search terms do not include specificities regarding elder care, it seems unlikely.

 My main concerns are as follows:

 1) Lack of clarity

 What is the focus of this review? Is it prescribing generally of prescribing in the context of elder care? This is not clear.

 2) Search strategy

 This lack of clarity impacts the search strategy. While search terms are sufficient for general prescribing, there are no specific search terms (or inclusion / exclusion criteria) regarding elder care.

 3) Overall

 I am slightly confused as the systematic review is already available as a preprint with a focus solely on prescribing in primary care (ie no mention of older people). So it seems that the study purpose has been changed but the protocol was not updated.

 These are the reasons why I approve this protocol with reservations as this issue needs to be clarified.

Is the study design appropriate for the research question?

Yes

Is the rationale for, and objectives of, the study clearly described?

Partly

Are sufficient details of the methods provided to allow replication by others?

Yes

Are the datasets clearly presented in a useable and accessible format?

Not applicable

Reviewer Expertise:

Dashboards, Data Visualisation, UX Design

I confirm that I have read this submission and believe that I have an appropriate level of expertise to confirm that it is of an acceptable scientific standard, however I have significant reservations, as outlined above.

# Response

**Published:** 2025-02-06

**Authors:** Patrick Moynagh

**Affiliations:** Department of General Practice, RCSI University of Medicine and Health Sciences, Dublin, Ireland

Many thanks for taking the time to review our manuscript. Your comments are very helpful and appreciated. Please see our reply below.

 We agree the focus of the systematic review lacks clarity in the introduction of the protocol. This systematic review aims to assess the effectiveness of interactive dashboards on prescribing for all patient cohorts within generalist primary care settings. The content in the introduction on older people and polypharmacy was to emphasise that medication-related harm is becoming a more pressing problem due to the aging population and rising levels of polypharmacy.

 However, our focus as described in the methods was and remains as all patient cohorts, and hence the search strategy does not include specific terms relating to older adults. This is consistent with the pre-registered PROSPERO protocol ( https://www.crd.york.ac.uk/prospero/display_record.php?RecordID=481475) and preprint, and thus there has been no change to the focus after commencing the systematic review.

 We have restructured the first paragraph of the introduction for clarity:

*“Reducing medication-related harm has been identified within the World Health Organisation’s (WHO) third global patient safety challenge in 2017, ‘Medication without Harm’. In the United States alone it is estimated that adverse drug reactions represent the third leading cause of death. While English data shows 1.5% of hospital admissions are as a result of an ADR. In Ireland ADRs are the third most common type of reported adverse event in the health care system. Most prescribing occurs in primary care and qualitative data from general practitioners (GPs) indicates prescribing has become more challenging, particularly for patients with multimorbidity and polypharmacy. Irish GPs in a nationwide cluster randomised controlled trial (RCT) evaluating a deprescribing intervention, viewed participation as an opportunity to review their prescribing practices and were interested in getting performance feedback. There is evidence that audit and feedback is effective in improving professional behaviour and that ongoing or repeated feedback is more effective.”*

# Reviewer report: Reviewer response for version 1

**DOI:** 10.21956/hrbopenres.15255.r41536

**Published:** 2024-12-26

**Authors:** Denis O'Mahony

**Affiliations:** 1 University College Cork, Cork, Ireland

This study protocol manuscript deals with a highly important issue i.e. preventable drug-related morbidity (PDRM) and the various interactive dashboard methods used in primary care settings. The protocol lays out clearly how the systematic review (SR) will be conducted, using an appropriate range of publicly available databases as well as other important sources for a thorough SR, including grey literature, trial registries and conference abstracts in order to avoid bias. The SR has already been registered on PROSPERO, the most internationally well-known registry for systematic reviews.

 The protocol is clearly written and concise and includes all of the elements that one would expect to find in a high-quality SR. I can find no intrinsic weaknesses in the protocol as described in this manuscript.

Is the study design appropriate for the research question?

Yes

Is the rationale for, and objectives of, the study clearly described?

Yes

Are sufficient details of the methods provided to allow replication by others?

Yes

Are the datasets clearly presented in a useable and accessible format?

Not applicable

Reviewer Expertise:

Polypharmacy in older people; clinical pharmacology

I confirm that I have read this submission and believe that I have an appropriate level of expertise to confirm that it is of an acceptable scientific standard.

# Response

**Published:** 2025-02-06

**Authors:** Patrick Moynagh

**Affiliations:** Department of General Practice, RCSI University of Medicine and Health Sciences, Dublin, Ireland

Many thanks for taking the time to review our manuscript. It is most appreciated.

# Reviewer report: Reviewer response for version 1

**DOI:** 10.21956/hrbopenres.15255.r43659

**Published:** 2024-12-09

**Authors:** Augustino Mwogosi

**Affiliations:** 1 The University of Dodoma, Dodoma, Dodoma Region, Tanzania

Summary of the Article

 The article outlines a systematic review protocol aimed at evaluating the effectiveness of interactive dashboards in optimizing prescribing in primary care. It also seeks to explore the characteristics of these dashboards to inform future developments in e-prescribing infrastructure. The protocol emphasizes the challenges of polypharmacy, multimorbidity, and inappropriate prescribing and positions interactive dashboards as innovative tools that combine real-time data visualization with feedback to improve prescribing practices.

 The systematic review will include interventional studies assessing interactive dashboards in primary care. The methods align with PRISMA-P guidelines, with a detailed plan for study selection, data extraction, and quality assessment. A narrative synthesis and potential meta-analysis are proposed to evaluate the findings comprehensively.

 Detailed Review

**1. Is the rationale for, and objectives of, the study clearly described?**

**Answer: Partly**

**Evaluation:**

 The rationale for the study is generally clear, highlighting the importance of addressing challenges in prescribing through innovative interventions like interactive dashboards. The protocol contextualizes the need for such tools by discussing their potential to integrate the strengths of CDSS and audit-and-feedback mechanisms.

 However, the link between the stated challenges (e.g., alert fatigue in CDSS, the transient impact of traditional feedback mechanisms) and the systematic review’s ability to address these gaps could be more explicitly developed.

 The objectives assessing the effectiveness and characteristics of interactive dashboards are clear but could benefit from additional specificity. For example, defining "effectiveness" and detailing how the characteristics will be described would strengthen the objectives.

**Suggestions for Improvement:**

1. Explicitly articulate how the systematic review will address the gaps identified in the rationale.
2. Provide operational definitions for key terms like "effectiveness" and clarify the scope of "characteristics" being explored (e.g., usability, integration, user engagement).

**4. Are the datasets presented in a usable and accessible format?**

**Answer: Partly**

**Evaluation:**

 While the protocol describes plans for data extraction and analysis, it does not provide examples or visualizations of how datasets or results will be presented in the review. Accessibility of data is implied but not explicitly addressed.

**Suggestions for Improvement:**

1. Include mock-up tables or figures to illustrate how extracted data or synthesized results will be presented.
2. Provide details on data-sharing plans, such as repositories or appendices where datasets will be made accessible.

General Comments

**Strengths**

- The protocol is methodologically robust, adhering to Cochrane and PRISMA-P standards.
- The systematic data extraction, quality assessment, and synthesis approach ensures reliability and transparency.
- The rationale addresses a significant issue in primary care, with interactive dashboards positioned as a promising solution.

**Limitations**

1. The rationale and objectives could be more explicitly linked to the research question.
2. The description of outcomes (e.g., "effectiveness") lacks specificity.
3. Plans for managing heterogeneity in the meta-analysis need to be more detailed.
4. Accessibility of data and presentation formats could be better clarified.

**Recommendations to Make the Article Scientifically Sound**

**Essential Points:**

1. link the rationale to the research question and specify how the review will address identified gaps.
2. Define key terms such as "effectiveness" and elaborate on how the characteristics of dashboards will be analysed.
3. Expand on handling heterogeneity in studies, including thresholds and criteria for conducting a meta-analysis.

**Optional Enhancements:**

1. Include mock-up tables or figures to illustrate how data will be presented.
2. Provide a clear plan for making datasets accessible through repositories or supplementary materials.

Is the study design appropriate for the research question?

Yes

Is the rationale for, and objectives of, the study clearly described?

Partly

Are sufficient details of the methods provided to allow replication by others?

Yes

Are the datasets clearly presented in a useable and accessible format?

Partly

Reviewer Expertise:

The core areas of research include Health Informatics and Decision-Support Systems, which focus on optimising the implementation and use of Electronic Health Record (EHR) systems to improve clinical decision-making in primary healthcare. Research also explores the integration of generative AI in EHR systems, particularly in low-resource settings.

I confirm that I have read this submission and believe that I have an appropriate level of expertise to confirm that it is of an acceptable scientific standard, however I have significant reservations, as outlined above.

# Response

**Published:** 2025-02-06

**Authors:** Patrick Moynagh

**Affiliations:** Department of General Practice, RCSI University of Medicine and Health Sciences, Dublin, Ireland

Many thanks for taking the time to review our manuscript and for your helpful comments. Please note our replies below. 

**Essential Points:**

**1) Link the rationale to the research question and specify how the review will address identified gaps.**

 We agree the rationale for this systematic review could be strengthened we have amended the first paragraph of the introduction as follows

*“Reducing medication-related harm has been identified within the World Health Organisation’s (WHO) third global patient safety challenge in 2017, ‘Medication without Harm’. In the United States alone it is estimated that adverse drug reactions represent the third leading cause of death. While English data shows 1.5% of hospital admissions are as a result of an ADR ^42^. In Ireland ADRs are the third most common type of reported adverse event in the health care system* *. Most prescribing occurs in primary care* *and qualitative data from general practitioners (GPs) indicates prescribing has become more challenging, particularly for patients with multimorbidity and polypharmacy* *. Irish GPs in a nationwide cluster randomised controlled trial (RCT) evaluating a deprescribing intervention, viewed participation as an opportunity to review their prescribing practices and were interested in getting performance feedback* *. There is evidence that audit and feedback is effective in improving professional behaviour and that ongoing or repeated feedback is more effective”.*

 Additionally the final paragraph of the introduction has been updated for clarity as follows *:*

*“In line with national and international campaigns to reduce medication-related harm and recent developments in e-prescribing infrastructure, this systematic review aims to explore the effectiveness of interactive dashboards in improving prescribing-related outcomes in primary care. These outcomes include potentially inappropriate prescribing (PIP) and drug utilisation rates (e.g., reducing prescribing volumes where lower rates are preferable or optimising prescribing patterns in line with guidelines). Additionally, it aims to describe the characteristics of these interventions to inform future intervention development and e-prescribing infrastructure.”*

***2)*** **Define key terms such as "effectiveness" and elaborate on how the characteristics of dashboards will be analysed.**

 Effectiveness is a commonly used in health services research to describe the extent to which an intervention achieves its intended objectives in real-world settings. For the purposes of this review, effectiveness will be assessed based on whether dashboards improve prescribing quality. These outcomes will be evaluated using the measures reported in the included studies, such as changes in prescribing rates or reductions in potentially inappropriate prescribing.

 Regarding the analysis of intervention characteristics, we will examine factors such the level of interactivity and whether the dashboards were delivered as part of a multi-faceted intervention or whether they included clinical decision support. We will explore how these characteristics relate to the reported outcomes, as detailed in the included studies.

 The introduction paragraph has been updated as follows:

*“In line with national and international campaigns to reduce medication-related harm and recent developments in e-prescribing infrastructure, this systematic review aims to explore the effectiveness of interactive dashboards in improving prescribing-related outcomes in primary care. These outcomes include potentially inappropriate prescribing (PIP) and drug utilisation rates (e.g., reducing prescribing volumes where lower rates are preferable or optimising prescribing patterns in line with guidelines). Additionally, it aims to describe the characteristics of these interventions to inform future intervention development and e-prescribing infrastructure.”*

**3) Expand on handling heterogeneity in studies, including thresholds and criteria for conducting a meta-analysis.**

 This review will be conducted in line with guidance set out in the Cochrane Handbook for Systematic Reviews of Interventions. As such we outlined that meta-analysis would be performed if there were sufficient studies examining the same outcome. To further clarify, we defined homogeneity based on several key factors:

**Study Design**: Studies needed to share a similar design (e.g., observational studies, interventional studies) to ensure comparability.

**Outcome Definition**: Outcomes needed to be defined and measured in a similar way across studies.

**Level of Measurement**: Outcomes measured at comparable levels, such as practice-level prescribing rates versus patient-level prescribing rates, were grouped separately to ensure consistency.

 These criteria aimed to balance comparability while maintaining a sufficiently broad scope to include a wide range of prescribing-related outcomes. Where studies differed in any of these aspects, meta-analysis was not performed, and results were synthesised narratively.

 We have updated the analysis section of the manuscript as follows:

*“We will narratively summarise the intervention types and those associated with improvements in prescribing outcomes. Additionally, we will narratively describe the prescribing related outcomes used by included studies. A quantitative synthesis (i.e. meta-analysis) will be considered if a sufficient number of homogenous studies are identified which examine the same outcome. We defined homogeneity based on several key factors, (i) study design (ii) outcome definition (iii) level of measurement.”*

*“As we believe that heterogeneity exists regardless of whether we happen to detect it using a statistical test, we will focus less on significance tests and instead further investigate the sources and impact of the heterogeneity (e.g. Risk of Bias) through sensitivity analysis.  In the event of substantial clinical or methodological heterogeneity, we will not report study results as pooled effect estimates and will synthesise study findings using the approach suggested in the Synthesis Without Meta‐analysis (SWIM) guidance.”*

**Optional Enhancements:**

**1) Include mock-up tables or figures to illustrate how data will be presented.**

 We will present extracted data using a modified TIDier checklist similar to Table 2. If meta-analysis is performed the results will be displayed graphically using a forest plot.

**2) Provide a clear plan for making datasets accessible through repositories or supplementary materials.**

 No new primary data will be collected for this study, as the review will synthesise publicly available data from published studies. All data generated or analysed during this study will be included in the published review and its supplementary information files. This will include the search strategies used for each database, details of the included/excluded studies, and any additional data extracted during the review process.
